# A Clinical Approach to Neuraxial Morphine for the Treatment of Postoperative Pain

**DOI:** 10.1155/2012/612145

**Published:** 2012-07-02

**Authors:** Borja Mugabure Bujedo

**Affiliations:** ^1^Department of Anesthesiology, Critical Care and Pain Medicine, Donostia University Hospital, 20014 San Sebastián, Spain; ^2^Pain Relief Unit, Acute and Chronic Pain Management, Donostia University Hospital, 20014 San Sebastián, Spain

## Abstract

Opioids are considered a “gold standard” in clinical practice for the treatment of postoperative pain. The spinal administration of an opioid drug does not guarantee selective action and segmental analgesia in the spine. Evidence from experimental studies in animals indicates that bioavailability in the spinal cord biophase is negatively correlated with liposolubility, and is higher for hydrophilic opioids, such as morphine, than lipophilic opioids, such as fentanyl, sufentanil and alfentanil. Epidural morphine sulphate has proven analgesic efficacy and superiority over systemically administered morphine for improving postoperative pain. However, pain relief after a single epidural injection of morphine could last less than 24 hours. Techniques used to administered and prolong opioid epidural analgesia, can be costly and inconvenient. Moreover, complications can arise from indwelling epidural catheterization, particularly in patients receiving anticoagulants. Clinical trials have shown that epidural morphine in the form of extended-release liposome injections (EREM) gives good analgesia for a period of 48 hours, with no need for epidural catheterisation. Intrathecal morphine produces intense analgesia for up to 24 hours with a single shot, and clinical recommendation is to choose the minimum effective dose and do not exceed 300 **μ**g to prevent the delay respiratory depression.

## 1. Introduction

Most surgical patients experience postoperative pain that persists for several days after surgery. Despite current pain-management guidelines, acute postoperative pain often remains undertreated and studies demonstrate that ineffective management can lead to poor clinical outcomes and serious adverse events. Effective pain control can contribute to several clinically valuable outcomes, including earlier patient mobilization and quicker recovery, which can result in a shortened hospital stay and reduced costs [1].

It is important to highlight that the treatment of postoperative acute pain requires a multimodality approach [2], whenever possible combining regional anaesthesia, analgesics that act centrally like paracetamol, others drugs that have a peripheral nonsteroidal anti-inflammatory effect (classic NSAID or selective ciclooxigenase-2 inhibitors), and opioids using different routes of administration, as well as coadjutant drugs, such as those for neuropathic pain like gabapentin or pregabalin. In the future, optimal analgesia should be based on clinical evidence for each surgical procedure [3] and should be combined with physiotherapy and rehabilitation programmes in order to minimise the period of postoperative recovery, hospital stay, and convalescence of our patients.

Opioids are considered a “gold standard” in clinical practice for the treatment of postoperative pain and morphine is one of the most common used opioids in the perioperative setting. Opioids are often added to neuraxial local anaesthetics (LAs) in patients undergoing surgery without general anaesthesia and in some institutions an opioid alone, typically morphine, is administered intrathecally as a single-dose injection before operation in patients undergoing major surgery under general anaesthesia [4]. It has been suggested that the optimal dose depends on the surgical setting and that there is a ceiling analgesic effect above which the risk of adverse effects outweighed the benefits of improved analgesia [5, 6], however, till now, the correct and safe epidural and intrathecal morphine dose has not been yet well established for many surgical procedures.

## 2. Clinical Use of Spinal Morphine

 The first published report on opioids for intrathecal anaesthesia belongs to a Romanian surgeon, who presented his experience using a mixture of cocaine and morphine in 1901, at Paris [7]. For nearly a century, spinal morphine was used without a known mechanism until 1973, when class of highly spinal specific opioid receptors were identified, and it was proven that direct application of morphine into the spine produced analgesia [8]. This became a reality when Wang et al. successfully used intrathecal morphine bolus dose injection in humans [9], and with the publication by Behar et al. in *The Lancet* in 1979, the first paper on the use of epidural morphine over 10 patients for the treatment of acute and chronic pain [10].

In the last 40 years, the scientific effort has been focused on identifying which types of opioids are suitable for spinal use and which are not. While spinal opioid administration can clearly be an effective analgesic technique, there is a widespread misconception that any opioid administered epidurally or intrathecally will produce analgesia by a selective spinal mechanism. This is simply not true, because multiples opioids (specially lipophilic) that are commonly administered spinally produce analgesia by uptake into the systemic circulation with subsequent redistribution to brainstem opioid receptors and, therefore, the analgesia produced is not superior to that produced by intravenous (IV) administration [11–13].

**Figure 1:**
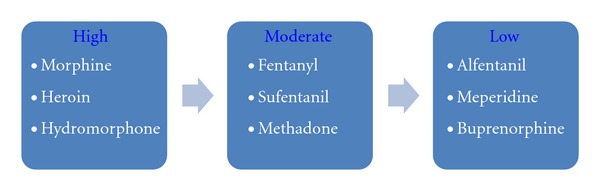
Spinal cord selectivity of neuraxial opioids in the treatment of acute postoperative pain [11–13].

 Bernards [12] carried out an elegant review of experimental studies in animals focusing on the measurement of opioid concentration in the epidural, intradural, spinal cord, and perispinal tissues, following spinal injection. He concluded that spinal opioid administration does not guarantee a spinal site of action and that available animal data clearly demonstrate that the spinal bioavailability of hydrophilic drugs (e.g., morphine, diamorphine, hydromorphone) is superior to that of lipophilic opioids (e.g., alfentanil, fentanyl, sufentanil, see Figure 1). Moreover, clinical studies confirm what one would predict from these animal studies: lipid-soluble opioids administered by continuous epidural infusion do not produce analgesia by a spinal mechanism [13–15].

However, intrathecally lipid-soluble opioids have a spinal site of action, but they are also cleared rapidly into plasma where they can redistribute to the brainstem producing significant early sedation and respiratory depression [12]. The most lipophilic opioids such as fentanyl and sufentanil are the opioids most studied and widely used intradurally in the context of postoperative pain given their rapid onset of action (10–15 min) and their short duration (2–5 h) [5, 11]. Several studies have focused on demonstrating the beneficial effect of the combination of lipophilic opioids with LA in ambulatory surgery and in the field of obstetrics as analgesic agents for labour pain [5, 15, 16]. In this way, the combination of fentanyl (20–30 *μ*g) or sufentanil (5–7.5 *μ*g) with bupivacaine or lidocaine leads to a faster onset of blockade and better intraoperative and immediate postoperative analgesia without increasing the degree of motor blockade or the time until discharge [16].

### 2.1. Epidural Morphine

**Figure 2:**
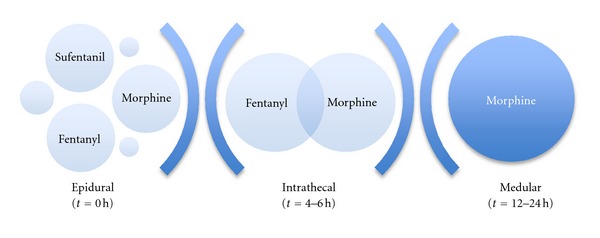
Spinal Bioavailability along the time of most common epidural administered opioids as a bolus [11–16].

(1) *Morphine *was the first opioid approved by the US Food and Drugs Administration (FDA) for spinal administration and it is the epidural opioid that has been the most widely used and with which others are compared [13]. Indeed, it could be considered the “gold standard” of spinal drugs, which does not imply the ideal one, as due to its spinal cord selectivity, the dose required is much lower for epidural than for parenteral administration (1/10), based on it presents the best spinal bioavailability (see Figure 2) [11–13]. It can be administered as a bolus (30–100 *μ*g/kg) or as continuous infusion (0.2–0.4 mg/h), which seems to induce better quality analgesia, and alone or together with local anaesthetics, as synergy between the drugs increases the overall analgesic effect [13, 15, 17].

 Epidural morphine sulphate (MS) has proven analgesic efficacy and superiority over systemically administered morphine for the treatment of postoperative pain [11, 13, 17–19]. However, pain relief after a single epidural injection of morphine could last less than 24 hours. Techniques used to administered and prolong opioid epidural analgesia, such as patient-controlled epidural analgesia (PCEA) pumps, continuous epidural infusion, and frequent reinjection, can be costly and inconvenient [20]. Moreover, complications can arise from indwelling epidural catheterization, particularly in patients receiving anticoagulants [21]. That is why, in 2004, the FDA approved the use of liposome-based extended-release epidural morphine (EREM) intended for single-dose administration by epidural route at the lumbar level, with mean duration of action of 48 h after a single injection use and delaying the peak concentration in the CSF until 3 hours after injection [22], without the problems associated with the catheter and with the expectation of improving on the overall failure rate with continuous epidural infusion technique which is estimated to be around 30% [13]. The basic points for its use include administration prior to surgery or after clamping the umbilical cord during a caesarean section and at least 15 minutes after the epidural test dose of LA and that no more epidural drugs be given for 48 h, since the continuous infusion of LA increases the release of morphine [23]. The formulation must not be injected through a filter as the particles may be disrupted.

In a randomized, controlled, dose-ranging study, Gambling et al. evaluated the analgesic efficacy of a single-dose extended-release epidural morphine over 504 patients after lower abdominal surgery [24]. Plotting a linear dose-response relationship to assess postoperative IV-PCA fentanyl consumption for breakthrough pain for 48 hours after surgery did the study. Comparing EREM (5, 10, 15, 20 25 mg) with 5 mg standard epidural morphine, pain relief was better at rest and with activity in all EREM groups with a significant overall reduction in intravenous fentanyl rescue use (*P* = 0.0002). As expected, the adverse effects reported were consistent with those of other epidural opioids (i.e., nausea, vomiting, pruritus, and hypotension) and were comparable between groups and well tolerated, with 97% rated as mild to moderate, with the exception of the significant differences observed in pruritus (*P* < 0.05) and urine retention (*P* < 0.05), both greater among the EREM groups. They concluded that single-dose EREM can provide up to 48 h of postoperative analgesia, but supplementation analgesia is still required in most patients.

In another review about epidural analgesia, based on two randomized, blinded studies of hip arthroplasty (*n* = 194, EREM dose 15–20–25 mg) and caesarean delivery (*n* = 75, EREM dose 5–10–15 mg) [25], Viscusi ER found that the rates of nausea and vomiting, pruritus, sedation, hypotension, pyrexia, headache, and urine retention were greater than 10%, while the risk of respiratory depression, peaking at 16 hours (only 0.6% occurred after 48 h.) was up to 4% (patients who received an opioid antagonist) with doses ≥20 mg and <1% with doses ≤15 mg. In another study [26], a meta-analysis approach was used to assess the adverse effects of EREM (*n* = 801) in comparison with IV opioids and standard epidural morphine. EREM 15 mg or greater was associated with a trend towards a higher incidence of hypoventilation (odds ratio: 0.48; 95% confidence interval [CI]: 0.21–1.09; *P* = 0.081; number-needed-to-treat (NNT) = 14) compared with placebo. The incidence of pruritus was significantly higher for all EREM doses compared with both placebo (*P* = 0.004) and standard epidural morphine (*P* = 0.03). Vomiting was also increased with EREM 15 mg or greater compared with placebo (odds ratio: 0.40; 95% CI: 0.18–0.89; *P* = 0.02; NNT = 5). Therefore, a multimodal analgesic regime was recommended to permit the use of lower EREM doses, thus reducing the risk for adverse effects and, accordingly, the latter dose (EREM ≤ 15 mg) has been accepted for clinical use. As with all opioids, the chief hazard is respiratory depression especially in elderly and debilitated patients and in those with compromise respiratory function. In a meta-analysis on the risk of respiratory depression compared to intravenous morphine in patient-controlled analgesia (PCA), an odds ratio (OR) of 5.80 (95% CI 1.05–31.93; *P* = 0.04) was estimated for the use of EREM [27].

In a review of five controlled clinical trials in knee replacement surgery, abdominal surgery and caesarean sections (*n* = 913), comparing IV PCA combined with epidural placebo, with a group receiving epidural administration of 5 mg of morphine sulphate (MS) and with another given EREM at various different doses (5 to 30 mg), it was demonstrated that the EREM group was the most effective at controlling postoperative pain after a single administration prior to surgery also as preventive analgesia. Pain at rest and with movement was thoroughly assessed at 10 time points within a 48-h period, as were any adverse effects, use of rescue analgesia, and the degree of patient satisfaction. The overall results lead to important conclusions, which could be transferred into clinical physician practice [28].

**Figure 3:**
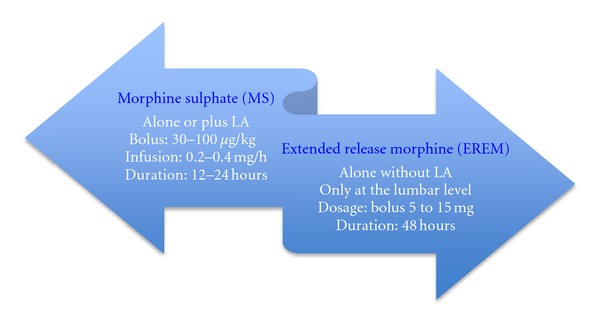
Recommended dosage for epidural morphine [13, 15, 17, 19, 23–28].

- All the groups using epidural morphine had higher levels of patient satisfaction despite a higher rate of adverse effects, reporting an “excellent” or “very good” level of satisfaction using fewer rescues IV PCA and rating their pain below the midpoint on the visual analogue scale for pain (VAS).
- Epidural morphine led to a lower use of IV rescue opioids than the group with epidural placebo and the effect was dose dependent. In the case of normal-release morphine, the difference was only significant for the first 24 h. On the other hand, the 10-mg EREM group experienced less pain during movement both 1 and 2 days after surgery compared to the 5-mg MS group, and used less IV rescue medication, though they did not have lower scores with respect to pain at rest.
- The use of epidural morphine causes a higher rate of pruritus, 15% of patients having moderate-to-severe itching, regardless of the formulation of morphine used, but no significant differences were found with respect to nausea or vomiting between the different treatment groups.
- They defined three treatments as relevant to clinical practice: IV PCA (plus epidural placebo in the trials), IV PCA plus 5 mg of epidural MS and IV PCA plus 10 mg of EREM. Overall, they recommended the combination of epidural morphine with an IV PCA regimen because this produced better analgesia than IV morphine/placebo group, and that there were also some advantages of using epidural 10 mg EREM over 5 mg MS. Their opinion was that dosing levels >10 mg were unlikely to be used in clinical practice.

(2) In conclusion, these controlled studies [23–28] have demonstrated that a single-dose EREM can provide up to 48 hours very good quality of postoperative analgesia with an acceptable and predictable side-effect profile. Prophylactic analgesia with EREM leads to a more satisfactory patient experience than IV PCA. Adequate pain prevention predicts patient satisfaction while the occurrence of unpleasant yet nonlife threatening adverse effects does not. Analysis of individual patient data from high-quality clinical trials provides important insights into the expected characteristics of new agents not immediately apparent from the original trials, and also informing thinking about improved clinical practice [28]. (Recommended dosage is summarized in Figure 3.)

 There are no publishing pharmacokinetic data on the administration of intrathecal EREM in humans. However, the vehicle for morphine in EREM consists of a liposomal compound known as Depofuam (Pacira Pharmaceuticals), which is also used in the administration of intrathecal chemotherapeutic agents. There are no description data to guide the treatment of a patient who receives intrathecal EREM, but Gerancher and Nagle [29] described a case of accidental spinal injection of 7.5 mg EREM in a 45-year-old women under an exploratory laparotomy and resection of an ovarian remnant, which was successfully treated without postoperative artificial ventilation, using an IV naloxone infusion (40–140 *μ*g/h) during 22 h until the patient reported any pain. She used 20 mg of IV-PCA morphine by the morning of postoperative day 2 and seemed to experience more somnolence initially, less analgesia subsequently, and a great deal more nausea than epidurally administered EREM.

### 2.2. Intrathecal Morphine

Following intrathecal administration, all opioids probably produce analgesia, at last in part, by a spinal mechanism [12, 13]. An opioid deposited intrathecally follows a multicompartmental pattern and simultaneously: it moves towards the head via the cerebrospinal fluid (CSF), undergoes spinal diffusion, binding to nonspecific receptors in the white matter as well as to specific receptors located in the grey matter, and undergoes clearance towards the epidural space, binding to the lipophilic structures therein, being distributed to the blood by vascular reuptake from these last two compartments [30]. The clinical characteristics of each opioid will be the consequence of the sum of all these types of distribution as they define its bioavailability and its spinal effect [31].

Hydrophilic opioid, such as morphine, crosses the blood-brain barrier slowly, bind to the epidural fat to a lesser extent, and more strongly to specific receptors in the grey matter, as well as having a slow plasma reuptake, maintaining concentrations in the CSF higher and for longer. As a result from this limited and slow transfer from the CFS, morphine presents a slow onset of action, extensive and prolonged rostral spread resulting in delayed respiratory depression (6–12 hours) and a broad band of analgesia surrounding the site of injection, and a relative long duration of action (18–24 hours) [5].

The qualification stems from data suggesting that lipophilic opioids, particularly sufentanil, produce analgesic plasma concentrations after intrathecal administration [11]. The relatively rapid movement of sufentanil into plasma to produce analgesic concentrations is responsible for the early respiratory arrests reported when this drug was administered intrathecally, occurring within the first 20–30 min after intrathecal injection [32, 33]. Perhaps the best clinical evidence of the limited ability of sufentanil to reach the spinal cord dorsal horn after intrathecal administration is the dose required producing analgesia. A common sufentanil dose is 10 *μ*g, which is equivalent to 10 mg of morphine based on their relative potency following IV administration. However, a typical intrathecal morphine dose is only 100 *μ*g, thus intrathecal administration results in a 100-fold decrease in the relative potency of morphine and sufentanil [12, 13].

Intrathecal opioid administration is an attractive analgesic technique since the drug is injected directly into the CSF, close to the structures of the central nervous system where the opioid acts. The procedure is simple, quick, and with a relative low risk of technical complications or failure. Intrathecal *morphine* without LA tends to be used as a single dose injection before operation, together with general anaesthesia to prevent pain after major surgery [4]. We should remember that its use in ambulatory surgery is not recommended and that the FDA has only approved an additive-free formulation for intrathecal use [5].

 In a meta-analysis [34] of 27 studies (15 concerning cardiothoracic, 9 abdominal, and 3 spinal surgery) on a total of 645 patients who received doses between 100 and 4000 *μ*g, it was demonstrated that among those given intrathecal morphine VAS at rest, on a scale of 10 cm, was 2 cm lower at 4 h and 1 cm lower at 12 and 24 h, and this effect was more pronounced with movement, the relative improvement being more than 2 cm throughout the period of monitoring. This lower score on a VAS was significantly better than the outcome with other analgesic techniques such as the administration of IV ketamine at low doses (scores fell by 0.4 cm), a regimen of postoperative NSAID (scores fell by 1 cm), and even continuous epidural infusion technique (scores fell by 1 cm), as assessed by the same authors previously [35]. The doses of opioids required intra- and postoperatively up to 48 h were lower among those given intrathecal morphine and the use of morphine up to 24 h was significantly lower in the abdominal surgery group (−24.2 mg, CI: −29.5 to −19) than the cardiothoracic surgery group (−9.7 mg, CI: −17.6 to −1.80). This more marginal benefit in the latter group makes the use of intrathecal morphine in thoracic surgery questionable, as a similar reduction in the amount of morphine required intravenously can be achieved using other strategies, such as the use of intraoperative ketamine (−16 mg/24 h) or postoperative NSAID (−10 to 20 mg/24 h) and even 4 mg of IV paracetamol may avoid the use of up to 8 mg of morphine in the first day after surgery [36]. The adverse effects were indeed more common in the group of intrathecal morphine with odds ratio of 7.8, 3.8 and 2.3 for respiratory depression, pruritus, and urine retention, respectively, although interestingly there was not a higher rate of nausea or vomiting. In the review, these authors did not detect a linear relationship between the dose administered and the degree of analgesia reached or any of the adverse effects. Accordingly, they were not able to recommend a minimum effective dose.

 In conclusion, in patients undergoing major surgery under general anaesthesia and receiving systemic opioids for break-through pain after operation, the additional use of intrathecal morphine decreases pain intensity, and also systemic morphine consumption, but does not decrease the risk of morphine-related adverse effects [13, 34]. The postoperative morphine-sparing effect is significantly weaker in patients undergoing cardiothoracic compared with abdominal surgery. The authors also concluded that we still do not know the optimal dose of intrathecal morphine when used alone [34].

 Nevertheless, in a meta-analysis based on studies on spinal anaesthesia [37], with morphine as adjuvant of an LA without general anaesthesia, the rate of adverse effects of intrathecal morphine was analysed (*n* = 790), compared to placebo (*n* = 524). A relationship was found between the dose of drug used and the occurrence of the adverse effects. Specifically, the group of morphine at low dose (*M* < 300 *μ*g) had a higher relative risk (RR) of nausea (RR 1.4, CI 1.1–1.7), of vomiting (RR 3.1, CI 1.5–6.4), and of pruritus (RR 1.8, CI 1.4–2.2), while the group of morphine at high doses (*M* ≥ 300 *μ*g) had a higher risk of pruritus (RR 5.0, CI 2.9–8.6) with similar values for the other parameters, compared to the placebo group. Further, the group given high doses had a higher rate of episodes of respiratory depression (7/80) than the low-dose group (2/247). The authors concluded that the use of intrathecal morphine at doses <300 *μ*g, although associated with a higher rate of adverse effects, is a safe dose, since among those on this dose the rate of episodes of respiratory depression was not higher than among the placebo group who received systemic opioids. The same authors, in a multicentre study involving 188 patients undergoing orthopaedic surgery [38], demonstrated that the use of rescue opioids was significantly lower for 72 h in a group given 200 *μ*g of intrathecal morphine than among those who received 100 *μ*g (*P* < 0.05) and in both groups with respect to the placebo group (*P* < 0.0001). The administration of intrathecal morphine was not associated with an increase in the number of episodes of respiratory depression, and moreover there was a significant increase in the number of patients, 70% of the group receiving 200 *μ*g of morphine, not requiring rescue medication for 48 h.

In another recent meta-analysis [39] about opioids added to LA for single-shot intrathecal anaesthesia in patients undergoing minor surgery, morphine (0.05–2 mg) and fentanyl (10–50 *μ*g) added to bupivacaine were the most frequently tested. Duration of postoperative analgesia was prolonged with morphine (weighted mean difference 503 min; 95% confidence interval [CI] 315 to 641) and fentanyl (weighted mean difference 114 min; 95% CI 60 to 168). Morphine decreased the number of patients needing opioid analgesia after surgery and decreased pain intensity to the 12th postoperative hour, and it also increased the risk of nausea (number needed to harm (NNH) 9.9), vomiting (NNH 10), urinary retention (NNH 6.5), and pruritus (NNH 4.4). With morphine 0.05 to 0.5 mg, the NNH for respiratory depression varied between 38 and 59 depending on the definition of respiratory depression chosen.

**Figure 4:**
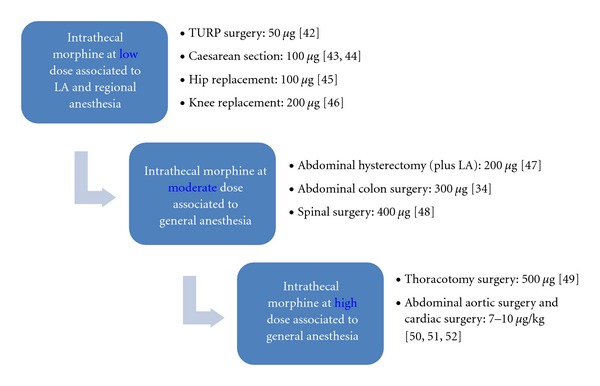
Recommended intrathecal morphine dosage for various surgical procedures [5, 13].

In a review by Rathmell et al. [5], about the use of drugs intrathecally for the treatment of acute pain, a maximum effective dose of morphine, beyond which the adverse effects seem to be higher than the benefits, was also recommended. In early trials with intrathecal morphine, doses ranged from 500–1000 *μ*g and profound sedation and respiratory depression were not uncommon [40]. In a carefully controlled study in healthy volunteers, prolonged respiratory depression was observed in all subjects who received 600 *μ*g of intrathecal morphine [41]. Recently, several trials have examined doses as small as 50 *μ*g, typically, extending only as large as 300 *μ*g, indeed, it appears that the efficacy of doses above this range is often limited by side effects like nausea, pruritus, severe urinary retention, and respiratory depression [5]. For this reason, they proposed a set of doses as a function of the surgical procedure, which are summarized in Figure 4.

It has been determined that the optimal dose of intrathecal morphine that produces satisfactory analgesia with minimum side effects in elderly patients undergoing transurethral resection of the prostate is 50 *μ*g [42]. The dose of 100 *μ*g produces analgesia comparable with doses as high as 400 *μ*g with significantly less pruritus (*P* = 0,0001) when combined with low-dose bupivacaine for caesarean delivery [43], and 100 *μ*g has been defined as the optimal dose in a qualitative and quantitative systematic review of randomized controlled trials for caesarean section [44]. Other clinical trials have demonstrated that the dose of 100 *μ*g of intrathecal morphine provides the best balance between efficacy and side effects, compared with doses of 50 and 200 *μ*g in older patients undergoing hip arthroplasty [45], as was the dose of 200 *μ*g optimal for knee replacement [46], in both cases associated to LA. In a very recent study, intrathecal morphine supplementation to bupivacaine reduced first 24 h PCA-morphine consumption after abdominal hysterectomy under general anaesthesia and found no benefit from increasing the dose over 200 *μ*g [47]. In a meta-analysis cited previously [34], 300 *μ*g was the most common used dose for abdominal surgery. It has also been demonstrated the efficacy of 400 *μ*g intrathecal morphine after posterior lumbar interbody spine fusion, as indicated by a significantly lower cumulate piritramide requirement versus the placebo group, without any serious increase of opioid associated side effects [48]. In a randomized, double-blinded comparison of intrathecal morphine (500 *μ*g), sufentanil (50 *μ*g), and their combination versus IV PCA morphine for postthoracotomy pain, both spinal opioids groups provided superior pain relief at rest (11 hours) and on coughing (8 hours) than did the IV PCA morphine group alone [49]. Greater doses have also been used associated to general anaesthesia after major surgery, up to 7–10 *μ*g/kg intrathecal morphine after aortic [50] or cardiac surgery [51, 52], but the overall benefits have not been well documented.

### 2.3. Practice Guidelines for the Management of Respiratory Depression Associated with Neuraxial Morphine Administration

 The most feared complication of opioid administration is respiratory depression. Ko et al. [53] reviewed the use of the term “respiratory depression” and found that there is no clear definition, leading to difficulty and confusion when comparing available studies. The incidence is infrequent for doses commonly used clinically but it is dose dependent for both hydrophilic and lipophilic opioids [54]. The incidence of respiratory depression associated with continuous epidural infusions containing opioids has been estimated from large observational studies, ranging from 0,09% to 0,4% [55–58]. Overall risk of respiratory depression after intrathecal o epidural opioids is less than 1%, and limited data suggest that it is similar to that of opioids delivered via parenteral route [59].

 Risk factors for the development of respiratory depression include large doses, concomitant use of additional opioids and/or sedatives, administration in opioid-naïve patients, and age >65 years [60]. Detection of respiratory depression after neuraxial opioids may be difficult. Respiratory rate may or may not decrease, and significant hypercapnia can occur despite a normal respiratory rate [6]. Pulse oxymetry may be valuable, but the most reliable clinical sign appears to be a depressed level of consciousness [40]. Protocols for monitoring vary, but typical duration of monitoring is 18 to 24 hours after spinal morphine and 4 to 6 hours after spinal fentanyl or sufentanil [6].

 The optimal neuraxial opioid dose is a balance between the conflicting demands of providing optimal analgesia while minimizing dose-related adverse effects. Dose-response studies show that neuraxial morphine appears to have an analgesic ceiling. The optimal “single shot” intrathecal dose appears to be 75–150 *μ*g and the ideal “single shot” epidural morphine dose is 2.5–3.75 mg [61]. Analgesic efficacy studies have not been adequately powered to show differences in the incidence of clinically significant respiratory depression. Opioid antagonists, such as naloxone, to prevent or treat opioid-induced respiratory depression have a number of limitations. Researchers have recently focused on nonopioid drugs such as serotonin receptor agonists and early evidence suggests that ampakine receptor modulators may be effective at reducing opioid-induced respiratory depression while maintaining analgesia [61].

 These are the recommendations from an update report made by the American Society of Anesthesiologists (ASA) Task Force on neuraxial morphine [62].

#### 2.3.1. Prevention of Respiratory Depression after Neuraxial Opioid Administration

- Particular attention should be directed toward signs, symptoms, or a history of sleep apnea, coexisting diseases or conditions (e.g., diabetes, obesity), current medications (including preoperative opioids), and adverse effects after opioid administration.
- Patients with a history of sleep apnea treated with noninvasive positive airway pressure should be encouraged to bring their own equipment to the hospital.

**(1) Drug Selection**

- Single-injection neuraxial opioids may be safely used in place of parenteral opioids without altering the risk of respiratory depression or hypoxemia.
- Single-injection neuraxial fentanyl or sufentanil may be safe alternatives to a single-injection of neuraxial morphine.
- When clinically suitable, extended-release epidural epidural morphine may be used in place of intravenous or conventional (i.e., immediate release) epidural morphine, although extended monitoring may be required.
- Continuous epidural opioids are preferred to parenteral opioids for anaesthesia and analgesia for reducing the risk of respiratory depression.
- When clinically suitable, appropriate doses of continuous epidural infusion of fentanyl or sufentanil may be used in place of continuous infusion of morphine or hydromorphone without increasing the risk of respiratory depression.
- Neuraxial morphine or hydromorphone should not be given to outpatient surgical patients.

**(2) Dose Selection**

- The lowest efficacious dose of neuraxial opioids should be administered to minimize the risk of respiratory depression.
- Parenteral opioids or hypnotics should be cautiously administered in the presence of neuraxial opioids.
- The concomitant administration of neuraxial opioids and parenteral opioids, sedatives, hypnotics, or magnesium requires increasing monitoring (e.g., intensity, duration, or additional methods).

#### 2.3.2. Detection of Respiratory Depression

 All patients receiving neuraxial opioids should be monitored for adequacy of ventilation (e.g., respiratory rate, depth of respiration assessed without disturbing a sleeping patient), oxygenation (e.g., pulse oximetry when appropriate), and level of consciousness.

(1) Patients receiving single-injection neuraxial hydrophilic opioids (e.g., morphine, hydromorphone, EREM not included).

- Monitoring should be performed for a minimum of 24 hours after administration
- Monitoring should be performed at least once per hour for the first 12 hours after administration, followed by monitoring at last once every 2 hours for the next 12 hours (from 12 to 24 h).
- After 24 hours, frequency of monitoring should be dictated by the patient's overall clinical condition and concurrent medications.

(2) Patients receiving sustained or extended-release epidural morphine (EREM).

- Monitoring should be performed at least once per hour for the first 12 hours after administration, followed by monitoring at last once every 2 hours for the next 12 hours (from 12 to 24 h).
- After 24 hours, monitoring should be performed at last once every 4 hours for a minimum of 48 hours.

(3) Patients receiving continuous infusion or PCEA with neuraxial hydrophilic opioids (morphine, hydromorphone).

- Monitoring**  **should be performed during the entire time the infusion is in use.
- Monitoring at last once every hour should be performed for the first 12 hours after initiation, followed by monitoring at last once every 2 hours for the next 12 hours.
- After 24 hours, monitoring should be performed at last once every 4 hours.
- After discontinuation of continuous infusion or PCEA, frequency of monitoring should be dictated by the patient's overall clinical condition and concurrent medications.

#### 2.3.3. Management and Treatment of Respiratory Depression

- Supplemental oxygen should be available for patients receiving neuraxial opioids, but routine use is not recommended because it may increase the duration of apneic episodes and may hinder detection of atelectasis, transient apnea, and hypoventilation. It should be administered to patients with altered level of consciousness, respiratory depression or hypoxemia, and continued until the patient is alert.
- Intravenous access should be maintained if recurring respiratory depression occurs. Reversal agents should be available for administration to all patients receiving neuraxial opioids and if necessary appropriate resuscitation should be initiated.
- Noninvasive positive-pressure ventilation may be considered for improving ventilatory status.

### 2.4. Practice Guidelines for Acute Pain Management Associated with Neuraxial Morphine Administration

Anesthesiologists who manage perioperative pain should use therapeutic options such as epidural or intrathecal opioids, systemic opioid PCA, and regional techniques after thoughtfully considering the risks and benefits for the individual patient. These modalities should be used in preference to IM opioids ordered, “as needed.” The consultants and ASA members also strongly agree that the therapy selected should reflect the individual anesthesiologist's expertise, as well as the capacity for safe application of the modality in each practice setting. Special caution should be taken when continuous infusion modalities are used, as drug accumulation may contribute to adverse events [17].

**Level of Evidence for Central Opioid Analgesia (Morphine): (Consult Original Reference for Description of Each Level of Evidence) [17].**

- Randomized controlled trials (RCTs) report improved pain relief when use of preincisional epidural or intrathecal morphine is compared with preincisional oral, IV, or intramuscular (IM) morphine (Category A_2_ evidence).
- Meta-analyses of RTCs report improved pain relief and increased frequency of pruritus in comparisons of postincisional morphine and saline placebo (Category A_1_ evidence); findings for the frequency of nausea and vomiting were equivocal (Category C_1_ evidence).
- Meta-analyses of RTCs comparing postincisional epidural morphine with IM morphine report improve pain relief and an increase frequency of pruritus (Category A_1_ evidence).
- Meta-analyses of RTCs report improved pain scores and a higher frequency of pruritus and urinary retention when postoperative epidural morphine is compared with IM morphine (Category A_3_ evidence); findings for the frequency of nausea and vomiting were equivocal (Category C_2_ evidence).
- Meta-analyses of RTCs report improved pain scores when epidural morphine combined with LA is compared with epidural morphine alone (Category A_1_ evidence). Findings for the frequency of nausea and vomiting and pruritus were equivocal (Category C_1_ evidence).
- Meta-analyses of RTCs report improved pain scores, greater pain relief, and a higher frequency of pruritus when epidural morphine combined with bupivacaine is compared with epidural bupivacaine alone (Category A_1_ evidence); equivocal findings are reported for nausea and vomiting (Category C_1_ evidence).

## 3. Conclusions

Opioids are the most potent centrally acting analgesic drugs for the treatment of pain. On the recent years, since the discovery of spinal opioid receptors, the use of spinal opioids has been adopted in clinical practice in the hope of producing intense segmental analgesia that was devoid of the dose-limiting side effects associated with systemic opioid administration. Experimental studies have demonstrated that, after their neuraxial administration, liposolubility is inversely proportional to their spinal selectivity, which is higher for morphine, than for other more lipophilic drugs, such as fentanyl and sufentanil.

Morphine could be the most suitable opioid for neuraxial administration in the context of acute postoperative pain because it provides a very good quality of epidural and intrathecal analgesia, but its long elimination time and its potential to cause delayed adverse effects limit its routine use and require careful selection of patients and vigilance protocols, and it is not recommended for ambulatory patients.

Epidural administration of morphine is an effective route for an effective drug. In spite of its efficacy, single-injection use is limited by its short duration of action relative to postoperative pain and adverse effects associated with higher doses. On the other hand, controlled studies have demonstrated that a single-dose extended-release liposomal injections (EREM ≤ 15 mg) can be provided up to 48 hours very good quality of postoperative analgesia with an acceptable and predictable side effect profile, with no need for epidural catheterisation, but more studies are needed to routinely use this drug in clinical practice.

After intrathecal administration without LA associated to general anaesthesia, morphine-sparing effect is more pronounced after abdominal than after cardiac-thoracic surgery, and despite 30 years of clinical research, we still do not know the optimal dose of intrathecal morphine when used alone. Intrathecal morphine decreases pain intensity at rest and on movement up to 24 h after major orthopaedic and abdominal surgery when combined with LA, with doses as low as 50–100 *μ*g, and the clinical recommendation is do not exceed 300 *μ*g to prevent the delay respiratory depression. Intrathecal lipophilic opioids, such as fentanyl and sufentanil, are a good choice for labour, delivery, and caesarean section and for the ambulatory setting.

 The optimal neuraxial opioid dose is a balance between the conflicting demands of providing optimal analgesia while minimizing dose-related adverse effects. The optimal “single shot” intrathecal dose appears to be 75–150 *μ*g and the ideal “single shot” epidural morphine dose could be 2.5–3.75 mg, for the first 24 hours after surgery.

 All patients receiving neuraxial opioids should be monitored for adequacy of ventilation (e.g., respiratory rate, depth of respiration), oxygenation (e.g., pulse oximetry when appropriate), and level of consciousness.
